# Integrating approaches for quality guideline development in LactaMap, an online lactation care support system

**DOI:** 10.1186/s12884-021-03775-9

**Published:** 2021-04-23

**Authors:** Melinda Boss, Jennifer Turner, Patrick Boss, Peter Hartmann, Douglas Pritchard, Rhonda Clifford

**Affiliations:** 1 School of Allied Health, The University of Western Australia, Crawley, Western Australia 6009 Australia; 2 PEB Consulting Pty Ltd, 69 Federation St, Mount Hawthorn, Western Australia 6016 Australia; 3 School of Molecular Sciences, The University of Western Australia, Crawley, Western Australia 6009 Australia; 4 School of Medicine, The University of Western Australia, Crawley, Western Australia 6009 Australia

**Keywords:** Lactation, Breastfeeding, Clinical practice guidelines (CPGs), AGREE II

## Abstract

**Background:**

Health professionals caring for women and infants experiencing difficulty with breastfeeding have reported deficiencies in evidence-based lactation knowledge. LactaMap is an online lactation care support system with more than 100 clinical practice guidelines to support breastfeeding care. Clinical practice guidelines support medical decision-making by summarising scientific evidence into systematically developed statements for specific clinical circumstances. Both common-sense and theory-based approaches have been used for guideline development and debate continues regarding which is superior. LactaMap clinical practice guidelines were created over the course of 5 years using a common-sense approach that was refined inductively. The aim of this study was to incorporate a theory-based framework approach into the methodology for ongoing update and review of LactaMap clinical practice guidelines.

**Methods:**

The Appraisal of Guidelines for Research and Evaluation (AGREE) II instrument was chosen as the framework-based approach to appraise LactaMap guideline quality. The study was conducted in two phases. The first phase appraised all 103 original LactaMap guidelines. The second phase appraised a subset of 15 updated LactaMap guidelines using improved methodology guided by phase 1, as well as 15 corresponding original (un-updated) guidelines.

**Results:**

Mean Domain scores for 103 LactaMap original guidelines were above 75% in 3 of the 6 AGREE II quality Domains and no mean Domain score rated poorly. Update of guideline methodology was guided by phase 1 appraisals. Improved documentation of methods relating to questions in the Rigour of Development Domain resulted in improvement in mean Domain score from 39 to 72%.

**Conclusions:**

This study showed that a theory-based approach to guideline development methodology can be readily integrated with a common-sense approach. Factors identified by AGREE II theory-based framework provided practical guidance for changes in methodology that were integrated prior to LactaMap website publication. Demonstration of high quality in LactaMap clinical practice guideline methodology ensures clinicians and the public can have trust that the content founded on them is robust, scientific and of highest possible quality.

## Background

Compared with other major organs such as the heart, brain, liver, lungs and kidneys, there has been limited basic research into the physiology and biochemistry of the human lactating breast [1]. Of the knowledge that does exist, much has not translated into the training of clinicians. Doctors report that they receive insufficient education for the knowledge and skills required to assist with the medical care of women and/or their infants experiencing difficulty with lactation [2–4]. Indeed, most health professionals who work with women of childbearing age, children and families have been identified as lacking in lactation education [5].

Integration of evidence-based knowledge has proven to be an effective strategy for standardising clinical care [6]. It is therefore not surprising that when evidence-based lactation education has been identified as lacking, inconsistent advice is a factor commonly reported by mothers to contribute to early weaning [5].

To facilitate the translation of scientific evidence to practice, a multidisciplinary group based at The University of Western Australia developed LactaMap, an online lactation care support system [7]. LactaMap was designed to deliver the lactation evidence-base needed to support doctors and other health professionals caring for women and term infants experiencing breastfeeding difficulty. One of the ways LactaMap does this is via the online delivery of clinical practice guidelines (CPGs). CPGs support medical decision-making by summarising the best scientific evidence available into systematically developed statements for specific clinical circumstances [8]. LactaMap contains CPGs that summarise care for more than 100 conditions known to pose potential risk to lactation. Examples of CPG topics include “Nipple Bacterial Infection”, “Low Supply After Secretory Activation”, “Sexual Abuse Survivors and Breastfeeding” and “Breastfeeding and COVID-19”.

It is not enough for guidelines to be created; they must also be used. One of the factors that influences uptake of CPGs by clinicians is evidence of quality in their development [9]. Guideline quality has been shown to vary widely, therefore good quality methodology for their development is important for uptake of the resulting recommendations [10]. LactaMap CPGs were created over the course of 5 years using an approach to development that was based on common-sense and then refined inductively with time [11]. A common-sense approach is defined as the application of a group’s shared tacit knowledge [12]. Debate continues regarding the superiority of common-sense versus theory-based approaches for optimising development of health interventions such as CPGs [13]. A disadvantage of common-sense is that the implicit assumptions made are often not clearly identified. Theory-based approaches help explain how and why an intervention may succeed or fail. As theory had not yet been considered, it seemed reasonable to incorporate strategies identified by theory towards a method that combined both. One type of theory-based approach is a determinant framework, which is used to identify factors believed or found to influence uptake of an intervention by end users (in this case doctors and other health professionals) [13]. The framework provides a strategy to follow for development methodology. Application of a systematic framework to development of maternal and perinatal health guideline methodology has previously proven useful in identifying areas for improvement [14, 15]. The aim of this study was to incorporate a theory-based framework approach for processes used for ongoing update and review of LactaMap CPGs. The objectives were:

- To assess LactaMap CPG methodology using the framework of a validated guideline appraisal tool (Appraisal of Guidelines for Research and Evaluation (AGREE) II instrument).
- To use these assessments to guide changes to methodology and reassess a subset of guidelines updated using this new methodology.

## Methods

Ethics approval, reference number RA/4/1/8546, for conducting this study was granted by The University of Western Australia Human Research Ethics Committee.

The AGREE II instrument was chosen as an appropriate, validated tool suitable for use by guideline developers with sufficient available time and resources to assess CPG methodology [16]. This theory-based instrument considers the theoretical construct of quality, which is defined in this context as, “the confidence that the biases linked to the rigour of development, presentation and applicability of a clinical practice guideline have been minimised and that each step of the development process is clearly reported” [17]. The AGREE II instrument provides a framework to guide the CPG development by classifying 6 theoretical Domains, which are used to identify individual characteristics important for guideline quality [12, 18].

These characteristics consist of 23 questions ranked on a Likert scale from 1 (strongly disagree) to 7 (strongly agree). These 23 questions are grouped into 6 quality Domains. In addition, there are two global assessment questions [19]. Each of the 6 Domains captures one of the following aspects of guideline quality: Scope and Purpose, Stakeholder Involvement, Rigour of Development, Clarity of Presentation, Applicability and Editorial Independence. Each guideline is required to be appraised by at least two raters [18].

**Fig. 1:**
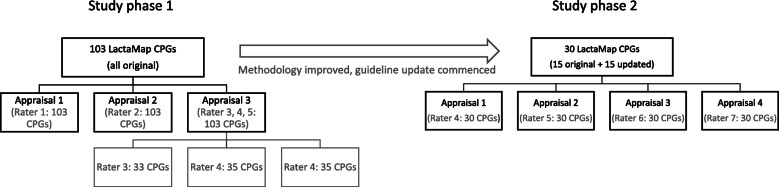
The 2 phases of LactaMap CPG appraisals

The study objectives were addressed sequentially in two phases. The first phase involved appraisal of the methodological quality of 103 original LactaMap CPGs. This was used to guide improvements to methodology developed using the common-sense approach. The second phase commenced a year later after a subset of 15 LactaMap CPGs had been updated using improved methodology guided by phase 1. A second group of raters appraised the updated LactaMap CPGs as well as the corresponding original (un-updated) version from the previous year (Fig. 1). Re-appraisal of original guidelines in phase 2 allowed comparison with appraisals conducted by the different raters in phase 1.

The AGREE II developers state that basic knowledge of evidence-based practice or health methodology is not required to use the instrument [18]. As recommended, each rater completed the online tutorials and practice exercise available from the AGREE enterprise website [20]. Appraisals were conducted independently, and raters did not discuss or view each other’s appraisal results until all appraisals were completed.

For the first phase, each of the 103 original LactaMap CPGs was independently assessed 3 times by 3 different raters from a pool of 5. The pool of raters consisted of 2 researchers and 3 Master of Pharmacy students, none of whom had been involved in LactaMap CPG development. Each pharmacy student appraised approximately one third of the 103 guidelines and collectively contributed one appraisal of each LactaMap CPG. The 2 researchers each appraised all 103 CPGs. Each of the 103 original LactaMap CPGs was therefore independently assessed 3 times by 3 different raters from a pool of 5 (Fig. 1). The second phase used 4 new raters, all Master of Pharmacy students who also were not involved in CPG development. The students in both phase 1 and 2 participated as part of their Pharmacy Practice Research Project unit. All raters provided written consent to participate. Strategies were specified in the ethics protocol to mitigate bias, which included administration of the participant information form and participant consent form by an academic who was independent from the study and had no involvement in the project. Students were advised that participation in the study was voluntary with no consequences associated with withdrawal at any time. Alternate research activities would be found for them for the Research Project unit should such withdrawal occur. All raters were encouraged to appraise CPGs critically, because the integrity of LactaMap depended on the identification of poor quality as well as high quality methodology.

Raters received a copy of the LactaMap Handbook, which provided information on methodology common to all LactaMap CPGs including scope and purpose, funding partners and procedures used [7]. Raters in the second year of the study were provided with the original handbook for original CPGs and an updated handbook for updated CPGs.

Appraisals were independently entered into a spreadsheet for each CPG by each rater and uploaded to an Access Database stored on the secure UWA Institutional Research Data Store (IRDS).

### Data analysis

Domain Scores were calculated by summing the individual question scores and scaling the total as a percentage of the maximum possible score for that Domain [18]:

(obtained score-minimum possible score) / (maximum possible score-minimum possible score). Descriptive analysis values reported were mean Domain Scores and median Item Scores across all LactaMap CPGs. The AGREE Consortium does not set patterns of scores to differentiate between high and low quality guidelines [18]. Instead it recommends that these decisions be guided by the context in which the instrument is being used. Increasingly, however, AGREE II users are applying a cut-off to distinguish between high and low-quality guidelines [21]. In line with previous studies, Domain Scores ≥75% and ≤ 50% were considered to be of high and low quality respectively [22, 23].

## Results

### AGREE II quality assessment

Mean Domain Scores from phase 1 appraisals are shown in Table 1. Three of the six mean Domain Scores rated as high quality (≥75%) and no mean Domain Score rated poorly (≤50%).

**Table 1:** Phase 1: Mean Domain Scores for 103 Original LactaMap CPGs

Domain	Phase 1 Mean Domain Scores^**†**^
	103 Original LactaMap CPGs^*^(SD^**^)
1. Scope and purpose	87% (3)
2. Stakeholder involvement	80% (4)
3. Rigour of development	54% (6)
4. Clarity of presentation	73% (7)
5. Applicability	66% (4)
6. Editorial independence	89% (1)

^†^Domain score = (obtained score-minimum possible score) / (maximum possible score-minimum possible score)

^*^High quality ≥75%, poor quality ≤50%

^**^Standard Deviation

The Mean Domain Score for Domain 3 (Rigour of Development) was only 4% above the upper limit for poor quality (50%). The median rating for individual questions in this Domain were used to identify specific areas where methodology was poor, indicated by a median rating less than 4 (Table 2). Median ratings less than 4 meant raters disagreed with a statement about LactaMap CPG quality. In phase 1 questions 7 and 8 resulted in median ratings of 2.

**Table 2:** Phase 1: Median Question Scores for 103 LactaMap CPGs

AGREE II Item number	Median Question Rating (Likert scale from 1 to 7)
**Domain 3. Rigour of Development**	**Phase 1 Original** (103 CPGs)
7. Systematic methods were used to search for evidence	2
8. The criteria for selecting the evidence are clearly described	2
9. The strengths and limitations of the body of evidence are clearly described	4
10. The methods for formulating the recommendations are clearly described	5
11. The health benefits, side effects, and risks have been considered in formulating the recommendations	6
12. There is an explicit link between the recommendations and the supporting evidence	5
13. The guideline has been externally reviewed by experts prior to its publication	6
14. A procedure for updating the guideline is provided	4
**Overall assessment question**	
1. Rate the overall quality of this guideline	5

In phase 2, a second group of raters appraised 15 of the original LactaMap CPGs that were created using the common-sense methodology and 15 corresponding updated LactaMap CPGs incorporating changes to methodology guided by phase 1. Mean AGREE II Domain Scores from phase 2 are shown in Table 3.

**Table 3:** Phase 2: Mean Domain Scores for 15 Original vs 15 Updated LactaMap CPGs

Domain	Phase 2 Mean Domain Scores^**†**^	
	15 Original LactaMap CPGs^*^ (SD^**^)	15 Updated LactaMap CPGs (SD)
1. Scope and purpose	92% (0)	95% (1)
2. Stakeholder involvement	75% (1)	80% (1)
3. Rigour of development	39% (2)	72% (2)
4. Clarity of presentation	73% (5)	82% (7)
5. Applicability	56% (3)	55% (3)
6. Editorial independence	94% (1)	94% (1)

^†^Domain score = (obtained score-minimum possible score) / (maximum possible score-minimum possible score)

^*^High quality ≥75%, poor quality ≤50%

^**^Standard Deviation

The Domains rated as high quality in phase 1were rated as high quality in phase 2. Domain 3 (Rigour of Development) was rated as poor quality in phase 1 and the original CPG’s in phase 2.

Median question ratings for the 15 original and 15 updated LactaMap CPGs are shown in Table 4. Median question ratings from phase 1 appraisals for the same 15 original LactaMap CPGs are also included for comparison with the original LactaMap CPGs appraised by raters in phase 2.

**Table 4:** Median Question Scores for 15 original (phase 1 and 2) vs updated (phase 2) LactaMap CPGs

AGREE II Item number	Median Question Rating (Likert scale from 1 to 7)		
Domain 3. Rigour of Development	Phase 1 original (15 CPGs)	Phase 2 origina (15 CPGs)	Phase 2 updated (15 CPGs)
7. Systematic methods were used to search for evidence	1	1	6
8. The criteria for selecting the evidence are clearly described	1	1	6
9. The strengths and limitations of the body of evidence are clearly described	4	2	3
10. The methods for formulating the recommendations are clearly described	5	5	6
11. The health benefits, side effects, and risks have been considered in formulating the recommendations	7	4	5
12. There is an explicit link between the recommendations and the supporting evidence	5	5	6
13. The guideline has been externally reviewed by experts prior to its publication	6	5	6
14. A procedure for updating the guideline is provided	4	3	6
**Overall assessment question**	**Phase 1 original** (15 CPGs)	**Phase 2 original** (15 CPGs)	**Phase 2 updated** (15 CPGs)
1. Rate the overall quality of this guideline	5	5	6

In phase 2 appraisals of original LactaMap CPGs, questions 7 and 8 again resulted in ratings less than 4. Additionally, Q9 and Q14 resulted in median question ratings of 2 and 3 respectively. Phase 2 appraisals of the updated LactaMap CPGs resulted in increases in all median question ratings in this Domain, with only Q9 scoring below 4.

The overall assessment question, which rates the overall quality of the CPG resulted in a median rating of 5 in both phase 1 and 2 appraisals of the original CPGs. This increased by 1 Likert point to 6 for the updated LactaMap CPGs, with a rating of 7 being the highest for overall guideline quality.

## Discussion

The AGREE II instrument provides a standard framework for assessing guideline development methodology so clinicians can be sure that recommendations made have been developed with low bias and high quality. The common-sense CPG development approach was already of high quality in Domains relating to Scope and Purpose, Stakeholder Involvement and Editorial Independence. The high score in the Scope and Purpose Domain indicated that LactaMap CPGs have clear objectives, clear descriptions of the health question covered and a specific description of the population to which the guidelines apply. The high quality score for Editorial Independence showed that the views of the funding organisation and any competing interests of the guideline development group did not influence guideline content. High quality for the Stakeholder Involvement Domain reflected the expertise in the multidisciplinary LactaMap guideline development group. This included international experts in human lactation research, experienced clinicians with expertise in breastfeeding as well as general practice (the target users) and consumer representation from the target patient population.

The first phase of the study successfully identified small changes where CPG methodology could be improved. These changes related to just two questions where raters disagreed with a statement about CPG quality. All were in the Rigour of Development Domain (Domain 3) and related to whether systematic methods were used to search for evidence (Q7) and whether there was a clear description of criteria for selecting evidence (Q8). This showed that while systematic methods were used to search for evidence, they were not well documented. Guideline developers were able to update the LactaMap Handbook so that processes were more transparent [7]. Following these changes, LactaMap CPG appraisals for Rigour of Development (Domain 3) in phase 2 showed the largest increase in mean Domain Score, from 39 to 72%.

In phase 2 appraisals of updated CPGs using the new updated methodology, only one question scored a median rating that indicated disagreement with the statement relating to quality in Domain 3. This question asked whether the strengths and limitations of the body of evidence are clearly described (Q9). This was expected as a decision had been made not to include such descriptions in the CPGs. Randomised controlled trials are considered to provide stronger evidence than cohort studies or observational research. Allocating a cohort to “no breastfeeding” is unethical and few studies continue long enough to determine any impact on long-term health outcomes known to be associated with breastfeeding [24]. The best evidence is sought for LactaMap CPGs, but until the quality of evidence is improved, individual citations will remain the source for users to assess.

The Applicability Domain, which appraises guideline dissemination and implementation tools and resources was not expected to score well in the AGREE II appraisals. The study was conducted under real-world conditions with LactaMap CPG creation and update occurring in tandem with development of the website. The LactaMap CPGs were appraised mid-way through this process and, while planned, considerations to facilitate implementation and dissemination were still in development. Despite this, the Applicability Domain did not score poorly, and its appraisal was important to ensure that all elements of quality guideline development methodology were captured for consideration prior to LactaMap website publication.

**Fig. 2:**
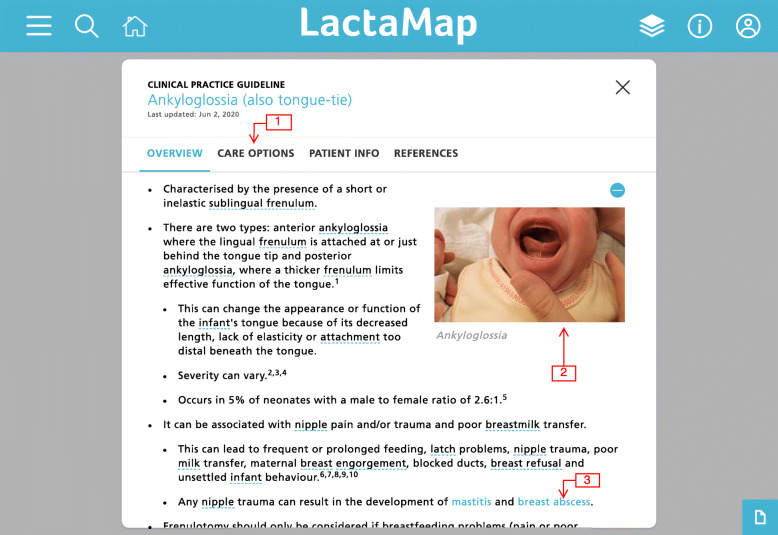
Example of LactaMap CPG as it appears online [7]. Indicating: 1. Content organised into tabs, 2. Image to assist diagnosis, 3. Blue highlighted text that links to other, related content

**Fig. 3:**
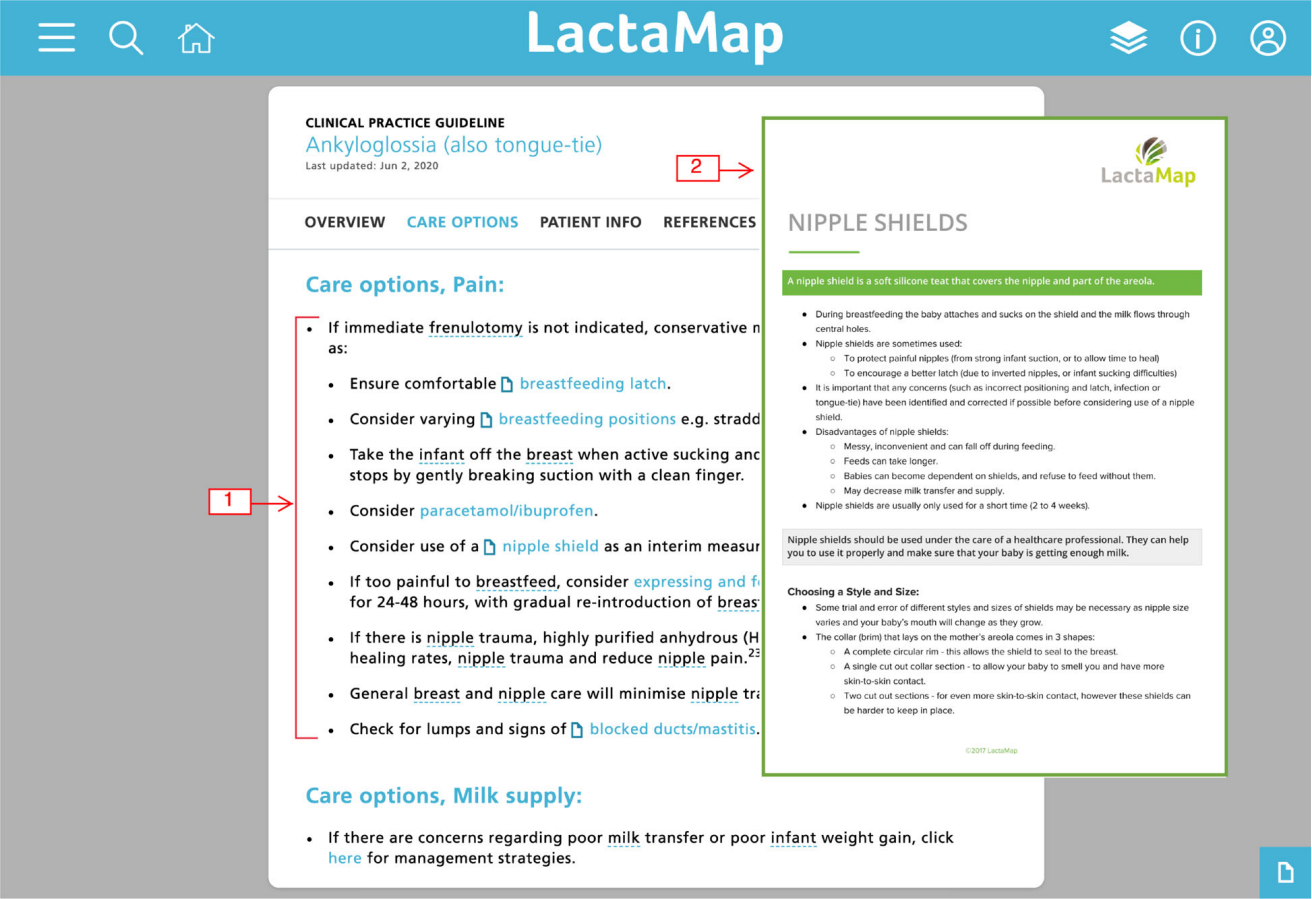
Example of LactaMap CPG showing content under the “Care Options” tab [7]. Indicating: 1. Different options for management and 2. Patient Information document that can be emailed or printed

Original LactaMap CPGs achieved a score of 73% for Clarity of Presentation (Domain 4), just outside the lower limit for high quality and this was higher than expected. LactaMap CPGs were intended to be accessed online with additional content available in linked documents, images to assist diagnosis (where available) and Patient Information documents designed to support care (Figs. 2 and 3). As the LactaMap website was not yet operational, appraisals were conducted on printed material that didn’t capture this dynamic online aspect of content presentation.

The clear and structured methodology resulting from this study proved very responsive when the need for evidence-based guidance regarding breastfeeding and COVID-19 (coronavirus disease 2019) emerged during the global viral outbreak. It supported rapid CPG delivery, with guidance published in LactaMap on the day the World Health Organisation declared a pandemic. Methodology has also supported ongoing flexible update of this Breastfeeding and COVID-19 guidance as new knowledge is published [25].

### Limitations

A limitation of the study is that raters in phase 2 were not blinded to whether a CPG was original or updated, however they were unaware that improvements in methodology had only been made to Domain 3 (Rigour of Development). The comparatively large improvement in this mean Domain score suggests that improvements to methodology were successful and were of practical use. Bias due to the potential for student raters to appraise CPGs favourably was mitigated by encouraging them to be critical.

The AGREE II instrument has been externally validated. Due to the pragmatic structure deployed for use of raters in phase 1 and the fact that methodology appraised for all LactaMap CPGs relating to three Domains (Editorial Independence, Scope and Purpose and Stakeholder Involvement) came from a common source (the LactaMap Handbook), statistical analysis of inter-rater reliability was not calculated.

## Conclusion

Common-sense based and theory-based approaches to CPG development each have advantages and disadvantages, with no single approach proven superior [12]. Combining both approaches allows consideration of real-world context derived from common-sense as well as explicit factors identified by theory as likely to influence outcomes. This study showed that a theory-based approach considering the construct of quality can be readily integrated with an existing common-sense based process for guideline methodology. The changes identified by applying the theory-based AGREE-II framework were able to be integrated prior to LactaMap website publication. Demonstration of high quality in LactaMap CPG methodology ensures clinicians and the public can have trust that the content founded on them is robust, scientific and of highest possible quality.

## Data Availability

The datasets used and/or analysed during the current study are available from the corresponding author on reasonable request.

## Abbreviations

AGREE: Appraisal of Guidelines for Research and Evaluation
CPG: Clinical Practice Guideline
ICC: Intra-class correlation coefficient
IRDS: Institutional Repository for Data Store
UWA: The University of Western Australia
COVID-19: Coronavirus disease 2019
